# Lumbar spinal ligament characteristics extracted from stepwise reduction experiments allow for preciser modeling than literature data

**DOI:** 10.1007/s10237-019-01259-6

**Published:** 2019-12-02

**Authors:** Nicolas Damm, Robert Rockenfeller, Karin Gruber

**Affiliations:** 1 MTI Mittelrhein, University of Koblenz-Landau, Universitätsstraße 1, 56070 Koblenz, Germany; 2 Mathematical Institute, University of Koblenz-Landau, Universitätsstraße 1, 56070 Koblenz, Germany

**Keywords:** Individual lumbar spine model, Biomechanics, Optimization, Muscle, Intervertebral disk, Facet joint

## Abstract

Lumbar ligaments play a key role in stabilizing the spine, particularly assisting muscles at wide-range movements. Hence, valid ligament force–strain data are required to generate physiological model predictions. These data have been obtained by experiments on single ligaments or functional units throughout the literature. However, contrary to detailed spine geometries, gained, for instance, from CT data, ligament characteristics are often inattentively transferred to multi-body system (MBS) or finite element models. In this paper, we use an elaborated MBS model of the lumbar spine to demonstrate how individualized ligament characteristics can be obtained by reversely reenacting stepwise reduction experiments, where the range of motion (ROM) was measured. We additionally validated the extracted characteristics with physiological experiments on intradiscal pressure (IDP). Our results on a total of in each case 160 ROM and 49 IDP simulations indicated superiority of our procedure (seven and eight outliers) toward the incorporation of classical literature data (on average 71 and 31 outliers).

## Introduction

Physiological ligament modeling is essential when aiming at accessing the force distribution or motion sequences within a lumbar spine. Multi-body system (MBS) as well as finite element (FE) modelers incorporates force–strain relations (characteristics) of the connective tissue to describe its behavior. It is widely accepted, based on experiments on isolated ligaments (Chazal et al. 1985; Nachemson and Evans 1968; Rissanen 1960; Tkaczuk 1968; Waters and Morris 1973) and functional units (Dumas et al. 1987; Myklebust et al. 1988; Panjabi et al. 1982; Pintar et al. 1992), that this force–strain relation consists of a nonlinear toe zone with a seemingly nonlinear–linear transition at higher strains. However, large variations between different data sets (see 4 Discussion) and thus the characteristics lead to unforeseeable variations in the model output as recently shown in Naserkhaki et al. (2018). Naturally, the question arises which ligament characteristics should be incorporated in order to account for preferably physiological behavior. We claim that characteristics obtained from recreating physiological experiments are superior to those obtained from isolated specimen.

In particular, Heuer et al. (2007) conducted a stepwise reduction experiment on a functional L4–L5 unit, where ligaments as well as other passive structures were gradually removed and the remaining segments underwent identical loading scenarios at each step, thereby measuring their ROM. They—as we will see correctly—assumed that this procedure ought to allow for model calibrations when conducted backward. Although originally thought for FE modelers, we are willing to accept this challenge in order to obtain ligament and disk characteristics for an MBS model of the lumbar spine. We are not aware of any prior attempts hereof.

Our model itself, as described in Sect. 2.2, consists of CT-based vertebral bodies transmitting forces mutually by intervertebral disks, facet joints, and ligaments. Nonlinear force–strain and torque–angle functions were set up for the ligaments and the intervertebral disk, respectively, and their parameters were estimated to fit experimental data (see Sects. 2.2.2, 2.2.4, and 3.1.2). To validate the characteristics obtained from the backward conducted stepwise reduction experiment, we proceeded in two stages:

First, we forward simulated the stepwise reduction experiment and compared the results to literature ligament characteristics. Here, we used four of the eight data sets presented in Naserkhaki et al. (2018), namely Chazal et al. (1985) (single ligament experiments), Shirazi-Adl et al. (1986) (literature synopsis from Adams and Hutton 1980; Farfan 1973; Nachemson and Evans 1968; Rissanen 1960; Tkaczuk 1968; Waters and Morris 1973), White and Panjabi (1990) (functional L4–L5 experiments from Panjabi et al. 1982) as well as Nolte et al. (1990) (ligament characteristics used by Schmidt et al. (2007), which had been based on Fig. 4.26 from Pingel (1991), which himself referred to Nolte et al. (1990)). The remaining data sets were (i) either referring to those before mentioned (Goel et al. 1995; McGill 1988; Rohlmann et al. 2006), (ii) did not account for a nonlinear characteristic (Goel et al. 1988; Myklebust et al. 1988; Pintar et al. 1992), or (iii) had an intransparent origin such as Goel et al. (1988), where no source or validation had been given.

Additionally, we evaluated the performance of an already existing MBS model from Rupp et al. (2015), see Appendix 1. Results showed a general superiority of our characteristics, cf. Section 3.1.2 and Appendix 2, which was, however, not surprising, because the characteristics were particularly tailored to stepwise fit the experimental data.

Hence, in a second stage, a wholly different experiment was simulated: the measurement of IDP between L4 and L5 after applying torques on the top segment of a lumbar spine (L2–S1) as conducted by Wilke et al. (1996). For this, we transferred our characteristics from the stepwise reduction experiment, as well as every other characteristic mentioned above, unchanged to a lumbar spine model. Additional to the passive structures, Wilke et al. (1996) had included wire cables to represent active skeletal muscle. In such an environment, the interplay between active and passive structures can be observed, increasing the physiological validity. Furthermore, the quantity of IDP can be both directly measured in vivo (Nachemson 1981; Rohlmann et al. 2001; Wilke et al. 1999, 2001) and obtained from modeling. As can be seen in Sect. 3.2 and Appendix 3, our model provided reasonable predictions of the IDP and outperforms the classical ligament data sets. Only the functional characteristics by Rupp et al. (2015), which are based on data from Chazal et al. (1985), yielded likewise satisfactory results.

## Data, model, and methods

### Experimental data

#### Stepwise reduction experiment

In their extensive experiment, Heuer et al. (2007) investigated the effects of various bending moments on the range of motion (ROM) within a functional L4–L5 unit, while gradually removing anatomical structures. The study included eight functional units, with an average age of 52 years that had been studied with a spine tester. Care had been taken to ensure that the intervertebral disks (IVD) had as little degeneration as possible. The functional units had been prepared such that in addition to the two vertebrae, only intervertebral disk, facet joints, and the ligamentous connections were present. Particularly, muscles had been removed completely. Step by step, the force-transmitting structures, i.e., the ligaments, the facet joints, and the nucleus, had been removed in the following order: supraspinous ligament (denoted w/o SSL), interspinous ligament (w/o ISL), flaval ligament (w/o FL), capsular ligament (w/o CL), vertebral arches (w/o VA), post. longitudinal ligament (w/o PLL), ant. longitudinal ligament (w/o ALL), nucleus pulposus (w/o NUC). Note that Heuer et al. (2007) denoted the CL by FC (facet capsules). Before each section step, pure moments of {0, 1, 2.5, 5, 7.5, 10} Nm had been applied along the three anatomical planes and the corresponding ROM had been measured.

The entirety of these experimental results, the mean ROM values from their Table 1 as well as the corresponding ranges from their Figs. 4, 5, 6, and 7, are hereinafter called *experimental data set one* (EDS1).

#### Intradiscal pressure experiment

In another classic experiment, Wilke et al. (1996) investigated the effects of various re-staged physiological loading conditions on the IDP within the L4–L5 segment of a lumbar spine (L2–S1). The study included seven lumbar spines, with an average age of 47 years that had been likewise studied with a spine tester. While the muscles had been completely removed, ligaments and bony tissue had been left intact. Yet aiming at a preferably physiological test environment, they added five pairs of symmetrical wire cables (80 N per pair), which ought to represent active lumbar spine muscles, particularly: m. multifidus with mm. rotatores in caudal direction, m. iliocostalis with longissimus, m. psoas major originating at both vertebrae and processus transversus, m. multifidus with mm. rotatores in cranial direction. The specimens underwent seven testing scenarios: (i) without any wire cables, (ii–vi) with each pair of wire cables separately, and (vii) with all wire cables acting jointly. In each scenario, the resulting IDP in the intervertebral disk (IVD) L4–L5 had been measured, while applying torques of ± 3.75 Nm to the uppermost vertebra (L2) along the three anatomical planes.

Since IDP can be measured both in vivo and in vitro and can also be calculated by modeling, this value is suitable for model validation. The entirety of these experimental results, as summarized in their Table 1, are hereinafter called *experimental data set two* (EDS2).

### A functional L4–L5 model

#### The vertebrae

**Fig. 1:**
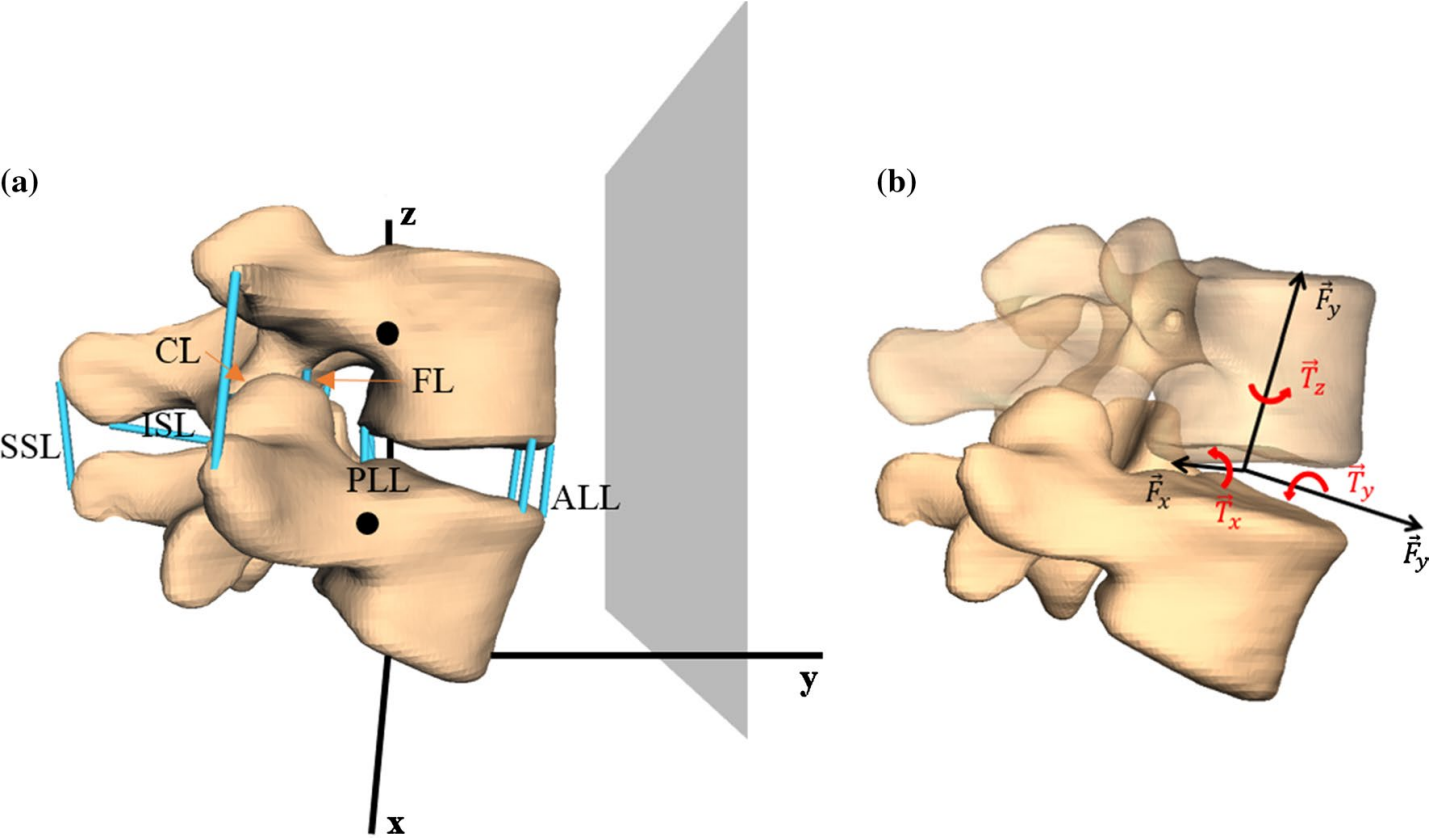
**a** Patient-specific, three-dimensional, L4–L5 MBS model, based on CT data including spinal ligaments (blue lines): ligamentum longitudinale anterius (ALL), ligamentum longitudinale posterius (PLL), ligamentum flavum (FL), ligamenta intertransversaria (ITL), ligamenta capsularia (CL), ligamentum interspinale (ISL), and ligamentum supraspinale (SSL). Big black dots indicate the centers of mass of the single vertebrae. Gray area represents the frontal plane that is dividing the body in a ventral and dorsal part and in our configuration lies parallel to the *x*–*z*-plane. **b** An example representation of the intervertebral disk (see Sect. 2.2.2), which acts as a force- and torque-transmitting joint (six degrees of freedom) between each pair of vertebrae

In order to reproduce the stepwise reduction experiment, a MBS model of a functional L4–L5 unit (and later the lumbar spine) was created on the basis of in vivo CT data of a whole human skeletal system. The vertebrae were manually segmented, ensuring that the position of the vertebrae to each other, the disk height, and the distances in the facet joints remained unchanged, see Fig. 1. Attention was also paid to a low degree of disk degeneration. The surfaces have no possibility for deformation. As already investigated by Dreischarf et al. (2010), this has a negligible effect on the results. The positions of the ligament insertion points were taken from the literature (Schünke et al. 2018) and checked by neurosurgeons from the University Hospital Mainz. The patient-specific vertebral surfaces resulted in individual insertion points and thus lever arms. All biomechanical multi-body simulations were conducted in the environment SimPack (Dassault Systèmes, Vélizy-Villacoublay, France).

#### The intervertebral disk

We assume the IVD to act as a translational and rotational spring–damper element, transmitting forces $F^{{\text{disc}}} = \left( {F_{x}^{{\text{disc}}}, F_{y}^{{\text{disc}}}, F_{z}^{{\text{disc}}} } \right)$, and torques $T^{{\text{disc}}} = \left( {T_{x}^{{\text{disc}}}, T_{y}^{{\text{disc}}}, T_{z}^{{\text{disc}}} } \right)$ dependent on deformation $r = \left( {r_{x} ,r_{y} ,r_{z} } \right)$ along and angle of rotation $\varphi = \left( {\varphi_{x} ,\varphi_{y} ,\varphi_{z} } \right)$ around the respective axes, as well as their corresponding time derivatives via

1 $$F^{{\text{disc}}} \left( {r,\frac{{{\text{d}}r}}{{{\text{d}}t}}} \right) = F_{{\text{spring}}}^{{\text{disc}}} \left( r \right) + d_{{\text{damp,tra}}}^{{\text{disc}}} \cdot \frac{{{\text{d}}r}}{{{\text{d}}t}},$$

2 $$T^{{\text{disc}}} \left( {\varphi ,\frac{{{\text{d}}\varphi }}{{{\text{d}}t}}} \right) = T_{{\text{spring}}}^{{\text{disc}}} \left( \varphi \right) + d_{{\text{damp,rot}}}^{{\text{disc}}} \cdot \frac{{{\text{d}}\varphi }}{{{\text{d}}t}}.$$

The nonlinear force–deformation curve $F_{{{\text{spring}},z}}^{{\text{disc}}} \left( r \right) = 690,234,060 \cdot r^{2} + 659748 \cdot r$ was determined from in vitro experiments (Damm 2019). The shear force characteristics $F_{{{\text{spring}},x}}^{{\text{disc}}}$ and $F_{{{\text{spring}},y}}^{{\text{disc}}}$ were adapted from the literature (Wilke et al. 2011). The damping coefficients were assumed to be $d_{{\text{damp,tra}}}^{{\text{disc}}} = 400,000 \frac{{\text{Ns}}}{{\text{m}}}$ and $d_{{\text{damp,rot}}}^{{\text{disc}}} = 100\;{\text{Nm}}\;{\text{s}}$. Note that damping is negligible in the equilibrium state experiments of Heuer et al. (2007) and Wilke et al. (1996). The rotational spring characteristics $T_{{\text{spring}}}^{{\text{disc}}} \left( \varphi \right)$ for flexion, extension, lateral flexion, and axial rotation were extracted from the penultimate reduction step in EDS1. Here, all ligaments had been removed with only the IVD remaining. We consequently applied the external torques from their Table 1 to our L4–L5 model and determined the internal torques necessary to reach the respective ROM. Thus, it could be assured that the IVD in simulation behaves exactly like the IVD in the experiment. We found, contrary to the common third-order polynomial approximation (Rupp et al. 2015), the likewise descriptive function

3 $$T_{{\text{spring}}}^{{\text{disc}}} \left( \varphi \right) = p_{1} \cdot \tanh \left( {\frac{{\varphi^{3} }}{{p_{2} }}} \right) + p_{3} \cdot \varphi$$

to suitably capture two data characteristics (cf. Fig. 4): (i) the occurrence of three visible inflection points and (ii) the beginning saturation of force at high absolute values of $\varphi$. A trust region algorithm was used in order to perform a least square fit and obtain the parameters $p_{1} ,p_{2} ,p_{3}$, see Table 1 and Fig. 4 in the Results section. Due to symmetry of the functional unit with respect to sagittal and transversal plane, positive and negative lateral flexion and axial rotation could be captured with the same characteristic (Eq. (3)), whereas the asymmetry in the frontal plane leads to the necessity of calculating two separate characteristic curves, one for flexion and one for extension.

**Table 1:** Parameter values, up to four significant digits, describing the nonlinear torque–angle characteristics of the IVD (Eq. (3): $T_{{\text{spring}}}^{{\text{disc}}} \left( \varphi \right) = p_{1} \cdot \tanh \left( {\frac{{\varphi^{3} }}{{p_{2} }}} \right) + p_{3} \cdot \varphi$) in the three anatomical planes, cf. Figs. 1 and 4

Direction of movement	*p*_1_	*p*_2_	*p*_3_
Flexion (rotation about *x*-axis)	10.67	0.005913	− 1.685
Extension (rotation about *x*-axis)	5.196	0.008417	24.50
lateral flexion (rotation about *y*-axis)	6.929	0.001482	19.76
Axial rotation (rotation about *z*-axis)	4.399	0.001141	42.38

In order to determine the IDP, we calculated the vertebrae’s cross-sectional area (CSA) and used the connection ${\text{IDP}} \approx 1.68 \cdot \frac{F}{\text{CSA}}$ found by Brinckmann and Grootenboer (1991), relating IDP to mean stress. This factor is in good congruence to other experimental findings, e.g., 1.5 from Nachemson (1960) and 1.3–1.82 from Dreischarf et al. (2013). Note that the latter source only gave the reciprocal values of 0.55 and 0.77, respectively.

#### Facet joints

**Fig. 2:**
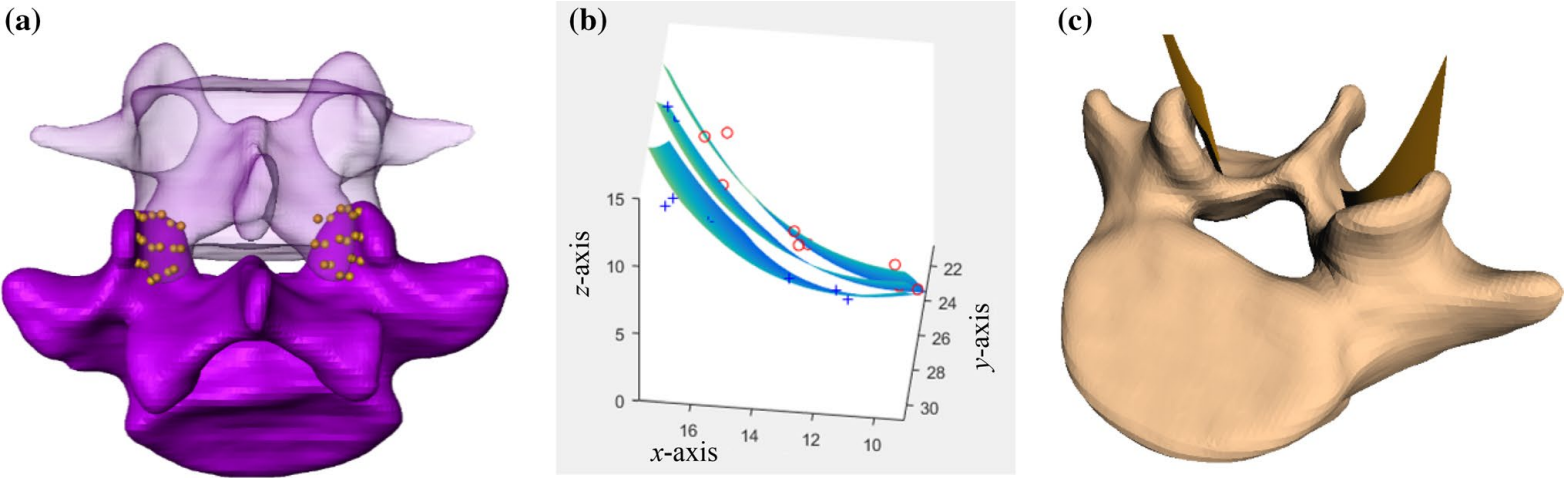
**a** Segment L4–L5 after segmentation. Nine landmarks were fixed on each processus articularis superior and inferior. **b** Resulting superior, inferior, and mean regression plane. The landmarks of the cranial vertebra are illustrated by the symbol red circle. The landmarks of the caudal vertebra are illustrated by the symbol blue +. (c) Vertebra L5 with the two calculated mean regression planes

The facet joint surfaces of the lumbar vertebrae seemingly have a convex curvature (Holzapfel and Stadler 2006; Peh 2011). We developed a computationally cheap method to preserve the individual curvature of the facet joint surfaces by means of a regression plane, see Fig. 2. We found that nine landmarks on each the processus articularis superior and inferior were sufficient to perform a stable fit of a convex cubic polynomial representing the surface area of an ellipsoid:

4 $$f\left( {x,y} \right) = p_{30} \cdot x^{3} + p_{03} \cdot y^{3} + p_{20} \cdot x^{2} + p_{02} \cdot y^{2} + p_{00} .$$

The resulting regression plane constitutes the mean of both superior and inferior regression planes, and the corresponding coefficients are shown in Table 2.

**Table 2:** Parameter values, up to four significant digits, describing the surfaces of the left and right facet joint between L4 and L5 (Eq. (4): $f\left( {x,y} \right) = p_{30} \cdot x^{3} + p_{03} \cdot y^{3} + p_{20} \cdot x^{2} + p_{02} \cdot y^{2} + p_{00}$)

Parameter	Left facet	Right facet
*p*_30_	− 0.007975	0.01011
*p*_03_	− 0.004237	− 0.008587
*p*_20_	− 0.1823	− 0.1236
*p*_02_	− 0.1734	− 0.2915
*p*_00_	47.31	44.26

Upon contact in the facet joint, contact forces always act perpendicular to the calculated regression plane in a spring–damper sense:

5 $$F^{{\text{fac}}} \left( {r,\frac{{{\text{d}}r}}{{{\text{d}}t}}} \right) = c_{{\text{stiff}}}^{{\text{fac}}} \cdot r + d_{{\text{damp}}}^{{\text{fac}}} \cdot \frac{{{\text{d}}r}}{{{\text{d}}t}}$$

Similar to Sect. 2.2.2, the stiffness $c_{{\text{stiff}}}^{{\text{fac}}} \approx 12,000\frac{{\text{N}}}{{\text{m}}}$ could be determined, since in a certain reduction step by Heuer et al. (2007) solely the facet joints were removed. The damping was further estimated as $d_{{\text{damp}}}^{{\text{fac}}} \approx 4000\frac{{\text{Ns}}}{{\text{m}}}$. With this method of facet joint modeling, the individual curvature of the joint surfaces can be preserved without increasing the calculation time, compared to a flat surface.

#### Ligaments

Our L4–L5 model contains all the ligaments that were dissected in the stepwise reduction experiment by Heuer et al. (2007) as well as the ITL as point-to-point force elements, see Sect. 2.1.1 and Fig. 1. Physiological prestrain $\varepsilon_{{{\text{lig}},0}}$ was taken into account on literature basis: 8% for ALL (Tkaczuk 1968), 10% for PLL (Tkaczuk 1968), 10% for FL (Nachemson and Evans 1968), 10% for ITL (Aspden 1992; Meijer et al. 2010), 10% for CL (Aspden 1992; Meijer et al. 2010), 4% for ISL (Robertson et al. 2013), − 6% for SSL (Robertson et al. 2013). In accordance with Gerritsen et al. (1995), ligaments were modeled as nonlinear spring–damper element dependent on strain $\varepsilon$ and velocity $\frac{{{\text{d}}\varepsilon }}{{{\text{d}}t}}$ via

6 $$F^{{\text{lig}}} \left( {\varepsilon ,\frac{{{\text{d}}\varepsilon }}{{{\text{d}}t}}} \right) = F_{{\text{spring}}}^{{\text{lig}}} \left( \varepsilon \right) + d_{{\text{damp}}}^{{\text{lig}}} \cdot \frac{{{\text{d}}\varepsilon }}{{{\text{d}}t}}.$$

It is well known that the force–strain curve of ligaments has a nonlinear toe zone with a linear transition (Chazal et al. 1985; Klein and Sommerfeld 2007, S.127; Shirazi-Adl et al. 1986; White and Panjabi 1990). We found a descriptive force–strain curve of connective tissue from Eq. (4) in Brown et al. (1996) to suitably represent this behavior:

7 $$F_{{\text{spring}}}^{{\text{lig}}} \left( \varepsilon \right) = a \cdot \ln \left( {e^{{\frac{\varepsilon + b}{d}}} + 1} \right) + c.$$

Similar to Eq. (5) from Rupp et al. (2015), we assumed the damping coefficient to be force dependent, but used a higher damping of $d_{{\text{damp}}}^{{\text{lig}}} = 10 \cdot F_{{\text{spring}}}^{{\text{lig}}}$ to avoid prolonged oscillations.

As suggested in the last sentence of the abstract in Heuer et al. (2007), a backward calibration yielded a least square fit of Eq. (7) similar to the procedure already explained in Sect. 2.2.2. The results of this fitting process are summarized in Table 3 and visualized in Fig. 5. Equation (7) was likewise fitted to data from Chazal et al. (1985), Shirazi-Adl et al. (1986), White and Panjabi (1990) as well as Nolte et al. (1990), respectively, in order to compare their performance to our approach. The optimized parameter tables and corresponding figures are shown in Appendix 1. Performance comparison of stepwise reduction and IDP experiments is given in Appendices 2 and 3, respectively.

**Table 3:** Parameter values describing the nonlinear force–strain ligament characteristics (Eq. (7):$F_{{\text{spring}}}^{{\text{lig}}} \left( \varepsilon \right) = a \cdot \ln \left( {e^{{\frac{\varepsilon + b}{d}}} + 1} \right) + c)$ within the backward performed stepwise reduction experiment, see Fig. 5 for corresponding graphs

	*a*	*b*	*c*	*d*
ALL	173.5	− 10.06	− 26.82	5.625
PLL	48.12	− 43.97	− 2.500	15.00
FL	47.11	4.210	54.85	− 11.25
CL	98.94	18.97	− 82.20	73.33
ISL	0.4517	− 3.502	0.0000	0.02414
SSL	1.218	− 22.97	0.0000	1.369
SSL neg.	− 50.49	− 36.00	− 0.02453	4.718

### A L2–S1 lumbar spine model

**Fig. 3:**
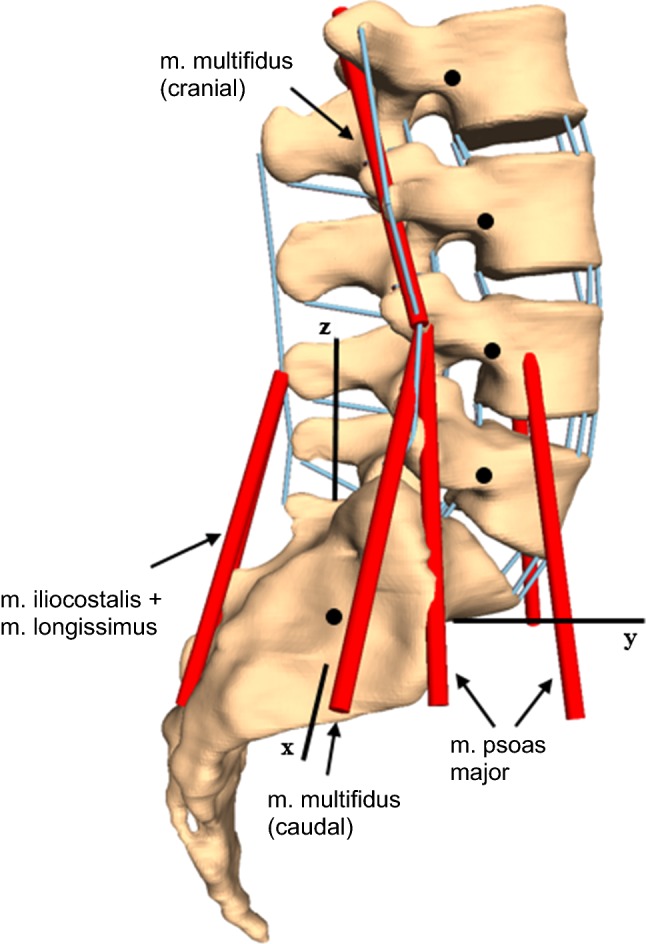
L2–S1 lumbar spine MBS model based on CT data including passive force-transmitting structures (IVD, ligaments (blue lines), facet joints) and the musculature represented by wire cables (red lines). Black dots represent the centers of mass of single vertebrae

So far, the passive force-transmitting structures, such as IVD, ligaments, and facet joints, were only developed for a functional L4–L5 unit. In order to recreate the IDP experiments from Wilke et al. (1996), we transferred the previously developed passive force-transmitting structures to a MBS model of a lumbar spine (L2–S1). The positions of the vertebrae to each other as well as the distances in the facet joints remained unchanged compared to in vivo data, see Sect. 2.2.1 and compare Figs. 1 and 3. Each IVD was modeled analogously to the L4–L5 disk from Sect. 2.2.2. The positions of the ligament insertion points were taken from the literature (Schünke et al. 2018) and checked again by neurosurgeons from the University Hospital Mainz. For each facet joint, individual regression planes were calculated according to the presented algorithm in Sect. 2.2.3. The already presented ligament modeling from Sect. 2.2.4 for the functional L4–L5 unit was also used throughout the full lumbar spine model. This rather generic scaling procedure could be improved, if stepwise reduction data from different levels of the lumbar spine were available.

#### Muscles

Wilke et al. (1996) had replaced muscles through symmetrical wire cables, transmitting a constant force of 80 N per pair. Therefore, one-dimensional, length-independent force elements were additionally integrated in the lumbar spine model, which transmitted constant traction toward the directional vectors given by Wilke et al. (1996). The following muscles were represented by wire cables in the experiment and thus included in our lumbar spine model:

- m. multifidus + mm. rotatores (caudal direction)
- m. iliocostalis + m. longissimus
- m. psoas major (origin: corpus vertebrae)
- m. psoas major (origin: processus transversus)
- m. multifidus + mm. rotatores (cranial direction)

Figure 3 shows the complete model setup.

## Results

### Stepwise reduction experiment

#### IVD and ligament characteristics

**Fig. 4:**
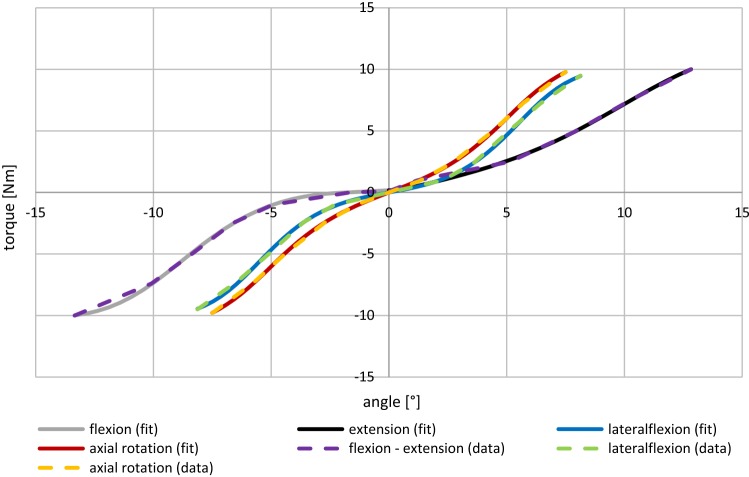
IVD torque–angle characteristics from fitting Eq. (3) (solid lines) to modeled data of our L4–L5 unit (dashed polygonal chain) recreating the penultimate reduction step (w/o ALL) from Heuer et al. (2007). Note that (i) due to vertebral asymmetry flexion and extension are modeled separately, (ii) a third-order polynomial formulation would neither account for the three visible inflection points nor the appearing torque saturation, and (iii) the angles are given in degrees, although calculations were conducted using radians. For the corresponding parameter values, see Table 1

**Fig. 5:**
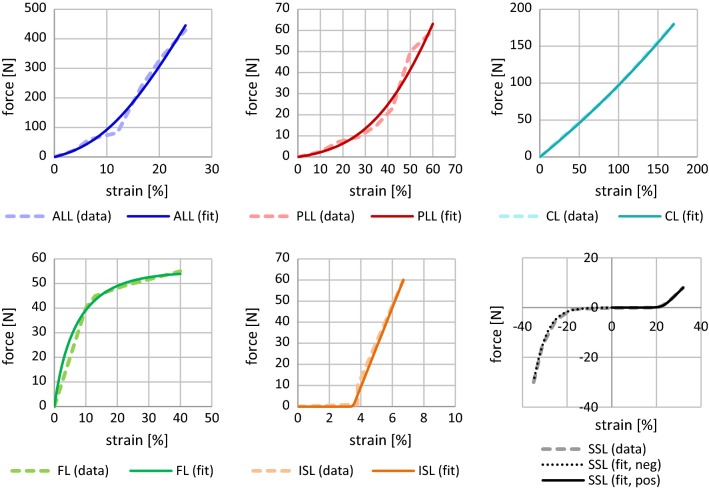
Ligament force–strain characteristics from fitting Eq. (7) (solid lines) to modeled data of our L4–L5 unit (dashed polygonal chain) recreating the reduction steps (from top left to bottom right: w/o PLL, w/o VA, w/o CL, w/o FL, w/o ISL, and w/o SSL) from Heuer et al. (2007). For the corresponding parameter values, see Table 3

To obtain IVD and ligament characteristics for our L4–L5 model, we performed the stepwise reduction experiment by Heuer et al. (2007) backward as suggested by the authors. Therefore, in a first step, the internal torque of the IVD without ligaments was determined iteratively, as described in Sect. 2.2.2. These data points were used to fit Eq. (3) and obtain the corresponding spring characteristics for flexion, extension, lateral flexion, and axial rotation. The optimized parameters are summarized in Table 1, with the resulting graphs as shown in Fig. 4. These results are in good congruence to characteristics reported in the literature (Karajan et al. 2013, Fig. 8a). In a second step, we added facet joint surfaces in terms of convex cubic polynomials, see Sect. 2.2.3. The resulting surface parameters for Eq. (4) are shown in Table 2. In the final step, we gradually added single ligaments and applied the given set of torques along all three anatomical planes, see Sect. 2.1.1 for the succession and torques as well as Sect. 2.2.4 for the prestrain values. Following this procedure, a characteristic curve for each ligament could be determined iteratively, by fitting Eq. (7) to the modeled data. Table 3 and Fig. 5 summarize the obtained parameter values and corresponding graphs.

#### Experimental data versus model output

**Fig. 6:**
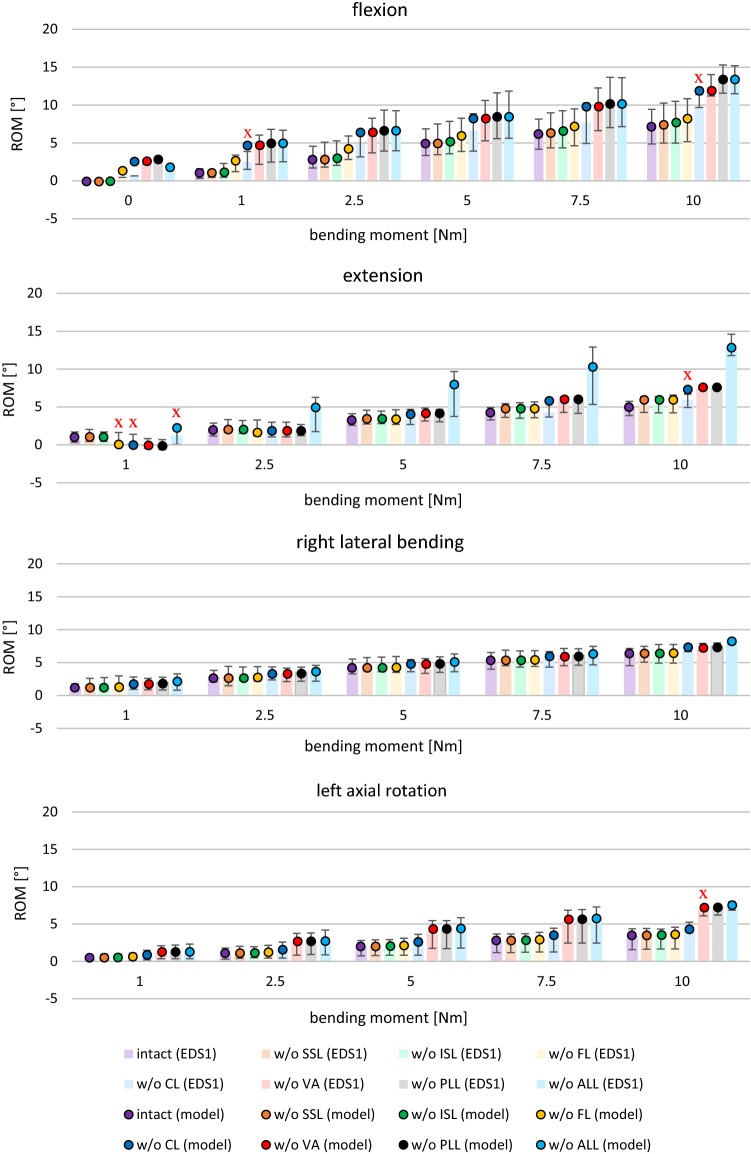
Bar plots visualizing the experimentally determined ROM of the stepwise reduction experiment by Heuer et al. (2007) at different torques (EDS1) along all three anatomical planes. Colored bars represent mean ROM values for different reduction steps. Black error bars indicate the ranges (minimum to maximum). The correspondingly colored circles show the simulation results of our L4–L5 model using the fitted IVD and ligament characteristics. Red crosses show the seven outliers, see Appendix 2. Note that there were no ranges given in the neutral position (0 Nm)

In order to validate the IVD and ligament characteristics found in the previous Sect. 3.1.1 as well as their interplay with our complete L4–L5 model (Sect. 2.2), we conduct a forward simulation of the whole stepwise reduction experiment, comparing the model output to EDS1 (Sect. 1.1). Figure 6 contains EDS1 in the form of a bar graph as well as the model output when applying the optimized IVD and ligament characteristics. Note that the last bar in each block (w/o ALL) was used to extract the IVD characteristics from Eq. (3) (see Table 1 and Fig. 4), whereas all other mean ROM values were used to extract the ligament characteristics from Eq. (7) (see Table 3 and Fig. 5).

Out of the 160 investigated scenarios (eight experimental stages times five different torques times four bending directions), only seven model output values appeared to be outliers from the experimental range, see Appendix 2. Taking this value of outliers as a benchmark, we compared the performance of our extracted ligament characteristics to prominent literature data. Therefore, we reran all 160 scenarios with the fitted ligament characteristics from Chazal et al. (1985), Shirazi-Adl et al. (1986), White and Panjabi (1990), Nolte et al. (1990) as well as modeled characteristics from Rupp et al. (2015), see Appendix 1 and Sect. 2.2.4. The results are likewise summarized in Appendix 2.

Apparently, any ligament characteristics from the literature that were extracted from isolated ligaments rather than physiological structures failed to reproduce certain loading conditions, in particular: The fitted ligament characteristics from Chazal et al. (1985) produced 57 outliers, giving no accurate estimate for flexion and extension movements. Similarly, simulation with data from Shirazi-Adl et al. (1986) led to 45 outliers, neither accurately reproducing flexion at intact units nor extension at reduced ones. Simulations with fitted ligament characteristics from White and Panjabi (1990) resulted in 94 outliers, failing to model flexion and extension movements as well as lateral bending almost completely. Using the fitted ligament characteristics from Nolte et al. (1990) yielded 110 outliers, failing to model all directions of movement. Finally, we implemented the ligament force–strain model from Rupp et al. (2015), which was also based on experimental data from Chazal et al. (1985), using their Eqs. (4) and (5) as well as Table 5. This model setup yielded 47 outliers mainly during flexion and extension movements.

### IDP experiment

In the prior Sect. 3.1, we found that the extracted IVD and ligament characteristics from EDS1 were more suitable in reproducing the stepwise reduction experiment than ligament characteristics fitted to isolated ligament data from the literature. This is, however, not surprising, since our IVD and ligament characteristics were extracted from this very experiment. To enhance the validation or our method, we aimed at recreating the results of an entirely different experimental setup, further extrapolating the L4–L5 unit to a lumbar spine (L2–S1). In the experiment by Wilke et al. (1996), the IDP in the L4–L5 IVD under different loading conditions had been measured after applying a torque of ± 3.75 Nm on the vertebra L2 along the three anatomical planes while fixing the sacrum (EDS2, see also Sects. 2.1.2 and 2.3). To account for near-physiological conditions, various wire cables had been spanned, representing active skeletal muscles.

**Fig. 7:**
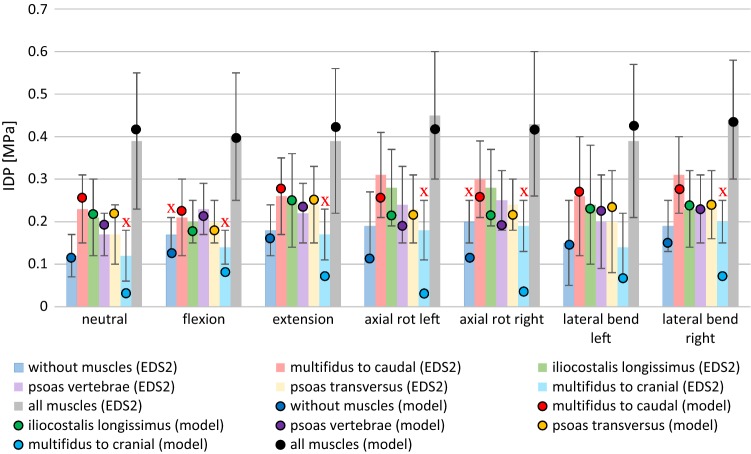
Bar plot showing experimentally determined IDP values (Wilke et al. 1996) in the L4–L5 disk under different loading conditions (EDS2). Colored bars represent the mean IDP values for different active muscle groups (wire cables): (i) none, (ii–vi) single muscle groups, and (vii) all muscles simultaneously. Black error bars represent the standard deviation. The correspondingly colored circles show our model output. Red crosses show the eight outliers, see Appendix 3

Figure 7 shows the resulting steady-state IDP values (mean ± SD) from all 49 scenarios (seven bending directions times seven muscle groups) as well as our model output, using exactly the previously extracted IVD and ligament characteristics from the stepwise reduction experiment. As can be seen in Fig. 7 and Appendix 3, our forward simulation missed the experimentally observed pressure interval seven out of 49 times; six of those in a single loading condition (multifidus to cranial). In this scenario, the ligaments were not able to sufficiently counteract the cranial traction forces.

The experiment was subsequently repeated with the already mentioned ligament characteristics from the literature to investigate their influence on the IDP. The results are also summarized in Appendix 3: With the characteristics of Chazal et al. (1985), there were 28 outliers, with those of White and Panjabi (1990), there were 41 outliers, with those of Shirazi-Adl et al. (1986), there were 47 outliers, and with those of Nolte et al. (1990), there were 27 outliers out of 49 loading conditions. Using the ligament modeling of Rupp et al. (2015), only 11 outliers were recorded.

## Discussion

Ligamentous structures play an essential role in spinal movement in terms of force-transmitting elements. Their full mode of operation thereby depends on the interplay with passive (e.g., IVD, vertebral bodies) as well as active (e.g., muscles) elements. In vivo loading conditions constitute for geometry-dependent alterations of lengths, lever arms, and thus force transmission. Hence, one would expect that ligament characteristics are examined and validated under physiological conditions. Yet, quite the contrary is the case; experimental data from single, cutout ligaments (Chazal et al. 1985; Nachemson and Evans 1968; Rissanen 1960; Tkaczuk 1968; Waters and Morris 1973), or functional units (Dumas et al. 1987; Myklebust et al. 1988; Panjabi et al. 1982; Pintar et al. 1992) are inserted unmodified into (FE and MBS) models to run simulations (Ayturk and Puttlitz 2011; Rohlmann et al. 2006; Schmidt et al. 2007).

An attempt to account for physiological validity was recently made by Naserkhaki et al. (2018) by comparing the performance of various ligament data sets in recreating experimental results. They found that the choice of ligament characteristics can cause certain variations on the model output, because of the large variation in the individual force–strain relations. We confirmed this observation, see our Appendices 2 and 3. For example, ligament data from White and Panjabi (1990) as well as Nolte et al. (1990) suggest very stiff behavior, whereas data from Shirazi-Adl et al. (1986) suggest pliable ligaments with a pronounced nonlinear toe region, see Appendix 1.

### Toward physiological ligament modeling

What are the reasons for the observed variance in ligament characteristics and what are the consequences for modelers?

In cadaver experiments, the individual ligaments are more or less strongly grown together, so that often no clear distinction between two different ligaments can be made. In this case, the dissecting person decides on the delineation of the respective ligament. But even if experimental setups are perfectly transparent, the direct transition to spine models is not recommendable; insertion points of ligaments are often planar, but must be reduced to a single point in the model. This can lead to significant length deviations between the dissected and the modeled ligament, particularly because the model vertebrae have different morphologies than the cadaver specimen. Moreover, the beginning and the end of some ligaments are hard to define, since some fibers of the ALL, PLL, and SSL pass over several vertebral bodies. The ligament itself runs along the whole spine. It should finally be noted that data sets are often not complete: The data set of White and Panjabi (1990) excluded the ITL and Chazal et al. (1985) left out the CL. These missing data should not be replaced by arbitrary data from other sources, due to our prior considerations.

Consequently, for modelers, the choice of particular data sets and individual insertion points can have unforeseen influences on the overall model output. This is why we suggest a line of action as proposed in this paper: First, the overall (patient-specific) geometry of the vertebrae, including IVD and ligament insertion points, is fixed. Second, own IVD and ligament characteristics are derived by recreating physiological experiments, accounting for the interplay of ligaments with each other as well as with active structures. Two of those experiments are the herein investigated stepwise reduction experiment by Heuer et al. (2007) and the IDP experiment by Wilke et al. (1996). Third, these data should be fitted with a differentiable function in order to smoothen the ligament behavior. The effect of this smoothening can be directly observed as data from Chazal et al. (1985) account for more outliers in our simulations than the respective characteristics from Rupp et al. (2015), which were based on this very data, see Appendix 1.

### Commendations and critics of the stepwise reduction experiments

In their comparative study, Naserkhaki et al. (2018) used recreation of stepwise reduction experimental data by Heuer et al. (2007) as a quality criterion. This is justifiable, because the experimental setup allows for full-range anatomical investigations, including most spinal ligaments, the IVD, and even the nucleus. However, Naserkhaki et al. (2018) did not take the consequently arising step: to follow the instruction at the end of the abstract from Heuer et al. (2007) and generate own ligament characteristics by modeling the experiment backward. In fact, to our knowledge, only one FE group accepted this challenge so far (Ezquerro et al. 2011). They obtained purely exponential, even more pliable ligament characteristics than we did in our Fig. 5 and did not validate the results against alternative characteristics or experiments. Thus, we presented here the first MBS-based demonstration of the practical feasibility of the procedure as well as its advantage toward implementing literature characteristics, see Figs. 4, 5, 6 and Appendix 2.

Despite their valuable methodology and data acquisition for calibrating lumbar spine models, we came across two points of criticism of the stepwise reduction experiments. First, the influence of the intertransverse ligament (ITL) (Behrsin and Briggs 1988) was not measured, which is why we could not derive a characteristic force–strain curve. Second and more severe, in their fifth reduction step, Heuer et al. (2007) removed the vertebral arches (w/o VA). This led to a significant increase (form 0.66 to 2.2 degrees) in the lordotic angle at 0 Nm torque, cf. their Table 1. In a more recent stepwise reduction experiment (Jaramillo et al. 2016), this particular case was not investigated. In their own discussion, Heuer et al. (2007) contributed this increase to a ventral shift of the center of mass. Yet, this reasoning does not agree with our observation. In our model simulation, removing the VA had only negligible effects on the lordotic angle. It had been mentioned that the VA were removed by sawing. We cannot assess the influence of this physical treatment on the relative vertebrae’s position, particularly, because no range of ROM was given at 0 Nm torque. As a direct consequence of this experimental shift that did not occur in our model, the modeled FL in the consecutive iteration step had to compensate for this mismatch, resulting in an unusual, concave force–strain relation.

### General applicability of physiologically obtained characteristics

So far, we addressed the validity of ligament characteristics in a forward simulation of stepwise reduction experiments within the L4–L5 segment, which were modeled backward to extract these very characteristics. Yet, it is not surprising that custom-tailored force–strain curves account for preciser modeling than arbitrary literature data. To emphasize the generality of the herein presented approach, we transferred the found characteristics, unchanged, to an entirely different loading condition: the change of L4–L5 IDP within a lumber spine containing constant force elements, representing ‘muscles’ (Wilke et al. 1996). Within this environment, our extracted ligament characteristics led to reasonable results, although not being specifically adapted to the new boundary conditions, cf. Fig. 7. The only outliers were found in the case of the cranially acting multifidus. We assume the reason hereof to be a combination of (a) a non-validated characteristic of ITL and (b) the inability of the modeled IVD to transmit traction forces. Finally, the superiority to classical literature data was shown by rerunning the simulation and finding that outliers in those cases were more of a normality than an exception, see Appendix 3. Solely, the model characteristics from Rupp et al. (2015) yielded similarly good results, indicating the advantage of smooth functions as mentioned above. However, the advantage of our model toward Rupp et al. (2015) is the patient-specific geometry of the vertebrae. Experimental data from Wilke et al. (1996) show that IDP developed differently in left and right lateral bending as well as axial rotation, thus implying an influence of individual morphology.

## Figures and Tables

**Table 4:**
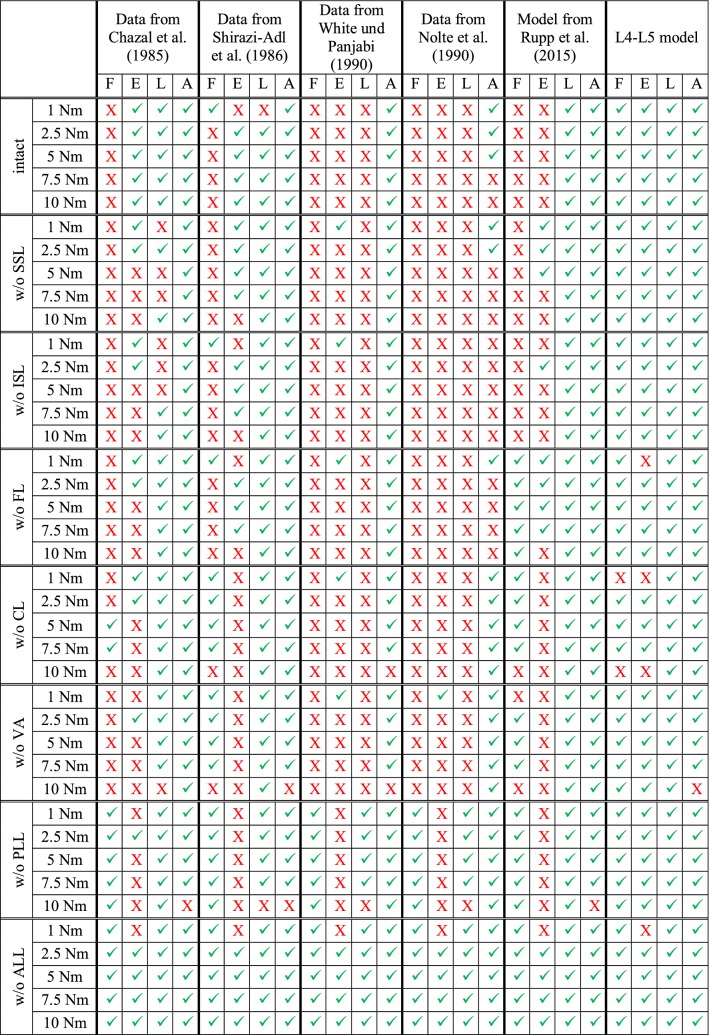
Synopsis of forward simulation results for stepwise reduction experiments. Red crosses indicate outliers of the experimentally determined range, and green checkmarks indicate congruence. For explanation of abbreviations, see text

**Table 5:**
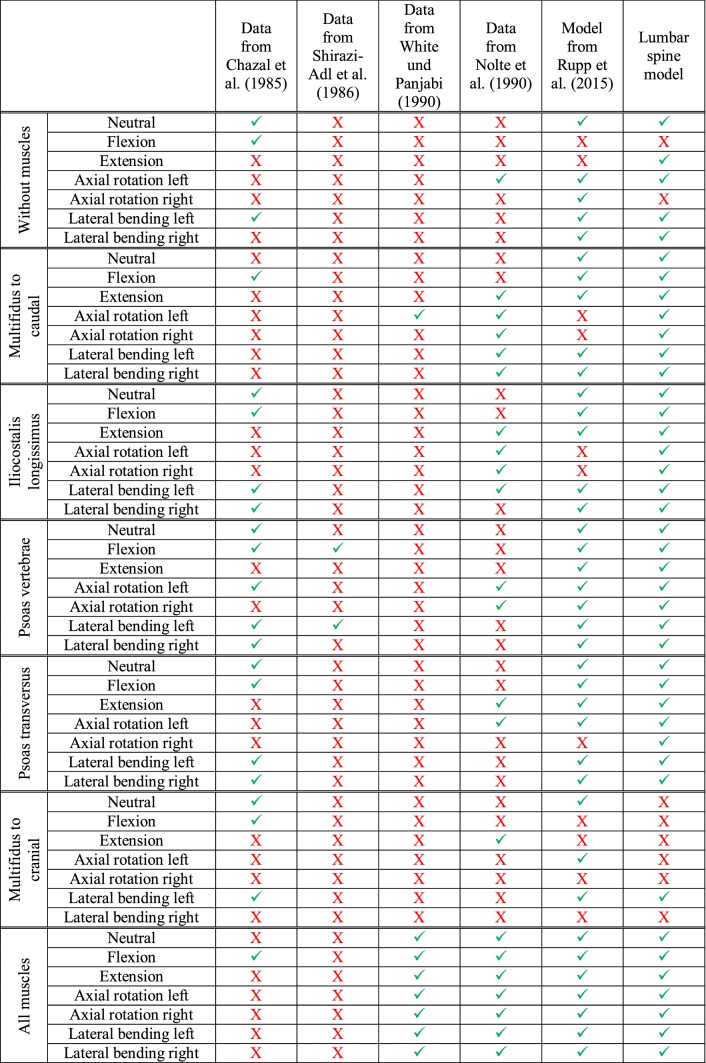
Synopsis of forward simulation results for IDP experiments. Red crosses indicate outliers of the standard deviation neighborhood of the experimentally determined mean values, and green checkmarks indicate congruence. For explanation of abbreviations, see text

## Appendix 1: Literature ligament characteristics

The herein shown figures contain data sets from Chazal et al. (1985), Shirazi-Adl et al. (1986), White and Panjabi (1990) as well as Nolte et al. (1990) (dashed polygonal chains), and the respective curve fits using Eq. (7) (solid lines). Resulting parameter values are shown in the accompanying table. Lastly, model characteristics from Rupp et al. (2015) are shown along with a description of the derivation of all necessary parameters.

### Data from Chazal et al. (1985)

For the curve fitting calculations, no force–strain measurements from the traumatic zone of a ligament were considered. Since the force–strain data of Chazal et al. (1985) consist of only three data points per ligament (cf. their Table 3), the curvature (d*) of the course was fixed here. Note that the CL had not been investigated.

### Data from Shirazi-Adl et al. (1986)

This data set, extracted from Fig. 5 of Shirazi-Adl et al. (1986), accounted for the most complete investigation and ought to be included in models based on literature data.

### Data from White and Panjabi (1990)

White and Panjabi (1990) had indicated in their Fig. 1–15 the ligament strength with respect to absolute length changes. Therefore, we calculated the respective strain using the presented in situ lengths in Fig. 3 of Panjabi et al. (1982). Since White and Panjabi (1990) do not specify a characteristic curve for the ITL, no parameters could be calculated.

### Data from Nolte et al. (1990)

Although determined from single ligament experiments instead of functional units, the data points extracted from Fig. 2 of Nolte et al. (1990) yielded ligament characteristics similar to White and Panjabi (1990), see above.

### Model from Rupp et al. (2015)

In their Eqs. (4) and (5), Rupp et al. (2015) presented a nonlinear-to-linear force–length ligament model, based on the previously mentioned data from Chazal et al. (1985). This model requires: (i) the in situ lengths $l_{{\text{lig,set}}}$ of all the ligaments in the model, (ii) number of point-to-point connections $n_{{\text{lig}}}$ representing one ligament, (iii) prestrain values $\varepsilon_{{{\text{lig,}}0}}$ from the literature, and (iv) force–strain tuples $\left( {l_{A} ,F_{A} } \right)$ and $(l_{B} , F_{B} )$ from Chazal et al. (1985). The ligament force $F_{{\text{el,lig}}}$ can then be calculated by

$$F_{{\text{el,lig}}} \left( {l_{{\text{lig}}} } \right) = \left\{ {\begin{array}{*{20}l} 0 \hfill & {l_{{\text{lig}}} < l_{{{\text{lig,}}0}} } \hfill \\ {K_{{\text{lig,nl}}} \cdot \left( {l_{{\text{lig}}} - l_{{{\text{lig,}}0}} } \right)^{{\nu_{{\text{lig,nll}}} }} } \hfill & {l_{{{\text{lig}},0}} \le l_{{\text{lig}}} < l_{{\text{lig,nll}}} } \hfill \\ {\Delta F_{{{\text{lig}},0}} + K_{{{\text{lig}},l}} \cdot \left( {l_{{\text{lig}}} - l_{{\text{lig,nll}}} } \right)} \hfill & {l_{{\text{lig}}} \ge l_{{\text{lig,nll}}} } \hfill \\ \end{array} } \right.,$$

where $\Delta F_{{{\text{lig}},0}} = \frac{{F_{A} }}{{n_{{\text{lig}}} }}$ denotes the nonlinear-to-linear transition force for one ligament strand and

$\Delta U_{{{\text{lig,}}l}} = \frac{{\Delta F_{{{\text{lig}},0}} }}{{l_{{{\text{lig}},0}} }} \cdot \frac{{l_{B} - l_{A} }}{{\frac{{F_{B} }}{{n_{{\text{lig}}} }} - \frac{{F_{A} }}{{n_{{\text{lig}}} }}}}$ the relative stretch in the linear part that results in force increase of $\Delta F_{{{\text{lig}},0}}$.

The length $l_{{\text{lig,nll}}} = \left( {1 + \Delta U_{{\text{lig,nll}}} } \right) \cdot l_{{{\text{lig}},0}}$ at which the transition occurs depends on the slack length $l_{{{\text{lig}},0}} = \frac{{l_{{\text{lig,set}}} }}{{1 + \varepsilon_{{{\text{lig}},0}} }}$ as well as the relative stretch of the nonlinear part $\Delta U_{{\text{lig,nll}}} = \frac{{l_{A} }}{{l_{{{\text{lig,}}0}} }}.$

The exponent $\nu_{{\text{lig,nll}}} = \frac{{\Delta U_{{\text{lig,nll}}} }}{{\Delta U_{{{\text{lig}},l}} }}$ as well as the nonlinear and linear spring constants $K_{{\text{lig,nl}}} = \frac{{\Delta F_{{{\text{lig}},0}} }}{{\left( {\Delta U_{{\text{lig,nll}}} \cdot l_{{{\text{lig}},0}} } \right)^{{\nu_{{\text{lig,nll}}} }} }}$ and $K_{{{\text{lig}},l}} = \frac{{\Delta F_{{{\text{lig}},0}} }}{{\Delta U_{{{\text{lig}},l}} \cdot l_{{{\text{lig}},0}} }}$, respectively, allows for continuous differentiability of the model.

The required input parameters as well as our calculated shape parameters are shown in the alongside table. Note that former are given in mixed fractions, since the spring constants are very sensitive to rounding errors particularly in $l_{A}$ and $l_{B}$, cf. values in Table 5 of Rupp et al. (2015), where the given accuracy is not sufficient to reproduce the parameter values.

## Appendix 2: Stepwise reduction ROM data: model vs. characteristic curves

Table 4 contains the synopsis of performances in the forward simulated stepwise reduction experiments. Each literature characteristic presented in Appendix 1 as well as our L4–L5 model underwent 160 scenarios, i.e., eight reduction steps (intact, w/o SSL, w/o ISL, w/o FL, w/o CL, w/o VA, w/o PLL, and w/o ALL) with five different torques (1 Nm, 2.5 Nm, 5 Nm, 7.5 Nm, and 10 Nm) in four bending directions (flexion [F], extension [E], lateral bending [L], and axial rotation [A]).

## Appendix 3: IDP data: model vs. characteristic curves

Table 5 contains the synopsis of performances in the forward simulated IDP experiment. Each literature characteristic presented in Appendix 1 as well as our L4–L5 model underwent 49 scenarios, i.e., seven experimental setups (without muscles, multifidus to caudal, iliocostalis longissimus, psoas vertebrae, psoas transversus, multifidus to cranial, and all muscles) with seven bending directions (neutral, flexion, extension, axial rotation left, axial rotation right, lateral bending left, and lateral bending right).
